# Obesity modulate serum hepcidin and treatment outcome of iron deficiency anemia in children: A case control study

**DOI:** 10.1186/1824-7288-37-34

**Published:** 2011-07-19

**Authors:** Mohammed Sanad, Mohammed Osman, Amal Gharib

**Affiliations:** 1 Department of Pediatrics, Faculty of Medicine, Zagazig University, Egypt; 2 Department of Biochemistry, Faculty of Medicine, Zagazig University, Egypt

**Keywords:** Obesity, Hepcidin, Iron deficiency, Children

## Abstract

**Background:**

Recently, hepcidin expression in adipose tissue has been described and shown to be increased in patients with severe obesity. We tried to assess the effect of obesity on hepcidin serum levels and treatment outcome of iron deficiency anemia in children.

**Methods:**

This was a case control study included 70 children with iron deficiency anemia "IDA" (35 obese and 35 non-obese) and 30 healthy non-obese children with comparable age and sex(control group). Parameters of iron status (Serum iron, ferritin, transferrin, total iron binding capacity and transferrin saturation) and serum hepcidin levels were assessed initially and after 3 months of oral iron therapy for IDA.

**Results:**

Compared to the control group, serum hepcidin was significantly lower in non-obese children with IDA(p < 0.01) and significantly higher in obese children with IDA (p < 0.01). Hepcidin increased significantly in non-obese children with IDA after 3 months of iron therapy (P < 0.01). On the other hand, obese children showed non-significant change in hepcidin level after iron therapy (p > 0.05). Although hepcidin showed significant positive correlations with Hb, serum iron and transferrin saturation in non-obese children with IDA, it showed significant negative correlations with Hb, serum iron and transferrin saturation in obese children with IDA (P < 0.05).

**Conclusions:**

Obesity increased hepcidin levels and was associated with diminished response to oral iron therapy in childhood iron deficiency anemia.

## Background

Obesity is associated with low-serum iron concentrations. The inverse relationship between iron status and adiposity was first reported in 1962, when Wenzel et al [1] unexpectedly found a significantly lower mean serum iron concentration in obese compared with non-obese adolescents. Most subsequent studies in pediatric and adult samples have shown similar results [2-5].

The etiology of the hypoferremia of obesity is uncertain. Among the proposed causes are deficient iron intake from an iron poor diet [2], and deficient iron stores owing to greater iron requirements in obese adults because of their larger blood volume [6]. Recently, fat mass was described as a significant negative predictor of serum iron and this hypoferremia seemed not to be explained by differences in iron intake [7].

Adipose tissue is a very active endocrine organ secreting numerous hormones and cytokines associated with important systemic effects on different metabolic processes [8]. Recently, hepcidin expression in adipose tissue has been described and shown to be increased in patients with severe obesity [9]. Hepcidin is a small, cysteine-rich cationic peptide produced by hepatocytes [10,11], secreted into plasma, and excreted in urine. Hepcidin expression is induced by iron stores and inflammation [11] and is suppressed by hypoxia and anemia [12]. Hepcidin is proposed to be a key regulator of iron metabolism and its discovery has changed our understanding of the pathophysiology of iron disorders [10]. Adipose tissue of obese patients produced increased amount of proinflammatory cytokines contributing to the development of a low-grade systemic inflammation in these patients [13].

At present, regulatory pathways that are generally thought to control liver hepcidin production include: (i) iron store-related regulation (ii) erythropoietic activity driven regulation, and (iii) inflammation related regulation. All are found to interact with liver cells to initiate the production of sufficient hepcidin for correct maintenance of iron homeostasis [14-17]. The aim of this study was to assess the effect of obesity on hepcidin serum levels and its relation to treatment outcome of iron deficiency anemia in children.

## Methods

This was a prospective case control study performed in Zagazig University Children Hospital and Outpatient Clinics in the same Hospital from April 2009 to August 2010. Informed parental consent was obtained for enrollment into the study. The study was done according to the rules of the local ethics committee of Faculty of medicine, Zagazig University. The study included 70 children with iron deficiency anemia [35 obese with BMI ≥ 95^th^centile for age and sex and 35 non-obese with BMI < 85^th^centile for age and sex]. 30 healthy non-obese children of comparable age and sex served as a control group.

Iron deficiency was defined as presence of one or more abnormal age-corrected iron parameters (iron, ferritin, transferrin and transferrin saturation). IDA was defined as concurrent iron deficiency and anemia [18].

We excluded from the study all patients with infections, collagen diseases, liver diseases, renal diseases, as will as children who received iron therapy in the previous three months.

### The following was done for patients and control group

1-Full history taking and detailed clinical examination including anthropometric measurements and body mass index (BMI) calculation.

2-Routine laboratory investigations including urinalysis, stool analysis, complete blood count (CBC) including blood indices, ESR and C-reactive protein (CRP).

3-Urine culture and sensitivity.

4-Liver function and kidney function tests.

5-Estimation of iron parameters (serum iron, ferritin, transferrin, total iron binding capacity (TIBC) and transferrin saturation).

6-Estimation of serum hepcidin-25 levels.

CBC, iron parameters and serum hepcidin-25 were re-evaluated for all children with IDA after three months-treatment course of anemia by oral ferrous sulfate (5 mg/kg/day of elemental iron).

### Serum hepcidin assay: Hepcidin C-ELISA

Before hepcidin analysis, sera were separated and stored frozen at 80°C at the Department of Biochemistry till the time of assay. According to the manufacturer's protocol; 96-well plates were coated with the antibody to human hepcidin and incubated with 100 μL (standard samples) or 200 μL (samples with very low concentration of hepcidin) of 1:20 dilution of serum in Tris-buffered saline containing 0.05% Tween-20 (TBS-Tween 20), with 10 ng/mL of biotinylated hepcidin-25 (Intrinsic LifeSciences, La Jolla, CA) added as the tracer. Standard curves were prepared by serial 2-fold dilution of synthetic hepcidin (Bachem Biosciences, King of Prussia, PA) 4000 ng/mL in TBS-Tween 20 buffer containing the tracer. The integrity and bioactivity of synthetic hepcidin and biotinylated hepcidin were verified by spectrometry and by bioassay with ferroportin-green fluorescent protein expressing HEK-293 cells [19]. After washing, the assay was developed with streptavidin-peroxidase and tetramethyl benzidine. The enzymatic reaction was stopped by sulfuric acid, and the plate was read at 450 nm on a DTX 880 microplate reader (Beckman Coulter, Fullerton, CA). Standard curves were fitted with 12-point fit using GraphPad Prism software (GraphPad Software, San Diego, CA). The fitted curve was then used to convert sample absorbance readings to hepcidin concentrations.

### Statistical analysis

SPSS for windows, version 11; was used for data analysis. Values were expressed as means ± SD. Chi-square test, Student t test and ANOVA test were used. Multiple comparison analysis by the least significant difference (LSD) was used. This test detects statistical difference between two means when ANOVA test refers to significances. Correlation between variables was assessed. P < 0.05 was considered significant.

## Results

There were non significant differences between the study groups as regards age, sex; p > 0.05, respectively (Table 1). BMI was significantly higher in obese children with IDA compared to both non-obese children with IDA and healthy control; p < 0,01, respectively (Table 1). Hb, MCV, MCHC, serum iron and transferrin saturation were all significantly lower in children with IDA (obese and non-obese) compared to the control, meanwhile there were non-significant differences between obese and non-obese children with IDA regarding these parameters; p > 0.05, respectively. Both TIBC and transferrin were significantly higher in children with IDA (obese and non-obese) compared to the healthy control, meanwhile there were non-significant differences between obese and non-obese children with IDA regarding TIBC and transferrin; p > 0.05, respectively (Table 1). Serum ferritin was significantly lower in non-obese children with IDA (7.52 ± 1.6 ng/ml) compared to both obese children with IDA (51.91 ± 8.7 ng/ml) and healthy control group (56.19 ± 4.1 ng/ml), (p < 0.05, respectively). However, a non-significant difference was found between serum ferritin in obese children with IDA and that in healthy control (P > 0.05) (Table 1).

**Table 1:** Baseline clinical and laboratory data of patients and control group.

	Obese children with IDA	Non obese children with IDA	Control	P
	(n = 35)	(n = 35)	(n = 30)	
**Age **(years)	6.96 ± 2.2	7.11 ± 2.57	7.11 ± 2.57	>0.05

**Male/female #**	22/18	23/17	11/9	>0.05

**BMI **(kg/m^2^)	25.70 ± 2.2 ^a^	16.30 ± 1.9 ^b^	16.2 ± 1.7 ^b^	<0.05

**Hb **(g/dl)	8.82 ± 1.2 ^a^	8.22 ± 1.4 ^a^	13.0 ± 1.4 ^b^	<0.05

**MCV **(fl)	70.80 ±4.6 ^a^	69.33 ± 4.0 ^a^	92.33 ± 7.0 ^b^	<0.05

**MCHC **(%)	26.3 ± 2.3 ^a^	25.6 ± 2.5 ^a^	33.7 ± 2.6 ^b^	<0.05

**Serum iron **(ug/dl)	28.83 ± 2.5 ^a^	29.28 ± 4.6 ^a^	90.45 ± 4.4 ^b^	<0.05

**TIBC **(mg/dl)	491 ± 24 ^a^	478 ± 15 ^a^	258 ± 15 ^b^	<0.05

**Transferrin saturation **(%)	5.87 ± 0.9 ^a^	6.13 ± 1.2 ^a^	35.05 ± 1.7 ^b^	<0.05

**Transferrin **(umol/L)	75 ± 7.1 ^a^	77 ± 6.8 ^a^	34 ± 2.3 ^b^	<0.05

**Ferritin **(ng/ml)	51.91 ± 8.7 ^a^	7.52 ± 1.6 ^b^	56.19 ± 4.1 ^a^	<0.05

**CRP **(mg/dl)	5.71 ± 0.7 ^a^	2.42 ± 0.5 ^b^	2.53 ± 0.6 ^b^	<0.05

**Serum hepcidin **(nmol/l)	4.96 ± 1.2 ^a^	0.11 ± 0.03 ^b^	1.61 ± 0.7 ^c^	<0.05

The P value is for ANOVA. a, b and c on means refer to significant difference between means when ANOVA test refers to significances by multiple comparison analysis (aa, bb, cc = non-significant, ab, ac, bc = significant). # Chi-square test.

Serum hepcidin was significantly lower in non-obese children with IDA (0.11 ± 0.03 nmol/l) compared to both obese children with IDA (4.96 ± 1.2 nmol/l) and healthy control group (1.61 ± 0.7 nmol/l); p < 0.01, respectively. Meanwhile, serum hepcidin was significantly higher in obese children with IDA compared to healthy control group; (P < 0.05), (Table 1).

CRP was significantly higher in obese children with IDA (5.71 ± 0.7 mg/dl) than in non-obese children with IDA (2.42 ± 0.5 mg/dl) and healthy control group (2.53 ± 0.6 mg/dl); P < 0.05, respectively, (Table 1). Meanwhile, there was no significant difference between non-obese children with IDA and control group as regard CRP; p > 0.05, (Table 1).

After three months of oral iron therapy, there were significant improvement of Hb, serum iron and TIBC in cases of IDA (P < 0.05), that was significantly more evident in non-obese children than in obese children (P < 0.05, respectively), (Table 2).

**Table 2:** Different clinical and laboratory parameters before and after iron therapy.

	Obese children with IDA	Non-obese children with IDA	P value
	(n = 35)	(n = 35)	
**BMI **(kg/m^2^)			
Baseline	25.70 ± 2.2	16.30 ± 1.9	<0.01
3 months after Fe therapy	25.91 ± 2.3	16.22 ± 1.2	<0.01
p value	>0.05	>0.05	

**Hb **(g/dl)			
Baseline	8.82 ± 1.2	8.22 ± 1.4	>0.05
3 months after Fe therapy	10.1 ± 0.8	12.8 ± 0.7	<0.05
p value	<0.05	<0.05	

**Serum iron **(ug/dl)			
Baseline	28.83 ± 2.5	29.28 ± 4.6	>0.05
3 months after Fe therapy	44.6 ± 6.9	65 ± 8.9	<0.05
p value	<0.05	<0.05	

**TIBC **(mg/dl)			
Baseline	491 ± 24	478 ± 15	>0.05
3 months after Fe therapy	385 ± 42	210 ± 23	<0.05
p value	<0.05	<0.05	

**Serum ferritin **(ng/ml)			
Baseline	51.91 ± 8.7	7.52 ± 1.6	<0.01
3 months after Fe therapy	52.19 ± 4.1	54.19 ± 4.1	>0.05
p value	>0.05	<0.01	

**Serum hepcidin **(nmol/L)			
Baseline	4.96 ± 1.2	0.11 ± 0.03	<0.01
3 months after Fe therapy	4.56 ± 1.4	1.62 ± 0.6	<0.01
p value	>0.05	<0.01	

P > 0.05 = non-significant, P < 0.05 = significant, P < 0.01 highly significant. (Student t test).

Serum ferritin increased significantly in non-obese children with IDA after 3 months of iron therapy; (P < 0.01). On the other hand, there was non-significant difference between serum ferritin before and after iron therapy in obese children (P > 0.05), (Table 2).

**Figure 1:**
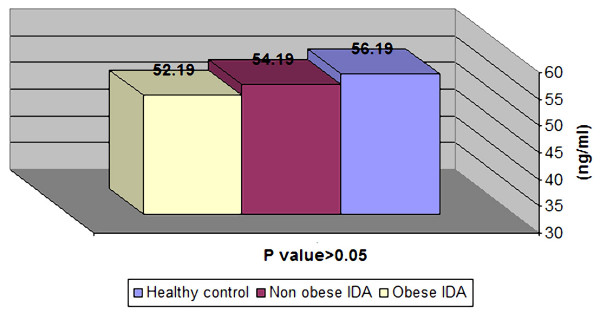
**Serum ferritin in healthy control and children with IDA after iron therapy**.

After oral iron therapy, there was non significant difference between patient groups and control group regarding serum ferritin levels (Figure 1).

Serum hepcidin increased significantly in non-obese children with IDA after 3 months of iron therapy (P < 0.01). On the other hand, there was non-significant change in serum hepcidin level after iron therapy in obese children (P > 0.05), (Table 2).

**Figure 2:**
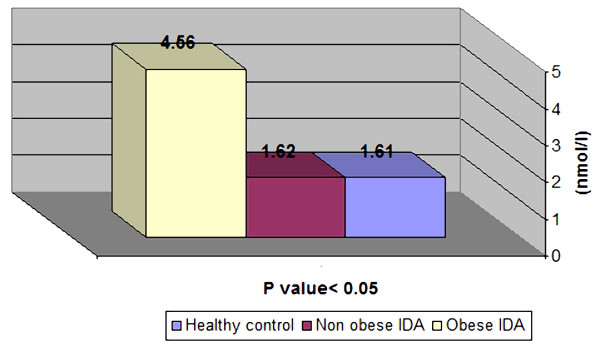
**Serum hepcidin in healthy control and children with IDA after iron therapy**.

Despite oral iron therapy, serum hepcidin levels in obese children persisted at significantly higher level than the control group. On the other hand, hepcidin levels in non-obese children were similar to that of the control (Figure 2).

In non-obese children with IDA, serum hepcidin showed significant positive correlations with Hb, serum iron and transferrin saturation (*r *= 0.498, *p *< 0.01, *r *= 0.478, *p *< 0.01, *r *= 0.468, *p *< 0.01, respectively), (Table 3). On the other hand, serum hepcidin showed significant negative correlations with TIBC and transferrin (*r *= -0.411, *p *< 0.05, *r *= -0.398, *p *< 0.05, respectively), (Table 3).

**Table 3:** Correlation between hepcidin and clinico-laboratory parameters in children with IDA.

	Serum hepcidin (nmol/l)			
	
	Non-obese children		Obese children	
	
	*r*	*p*	*r*	*p*
**Age **(year)	0.235	>0.05	0.122	>0.05

**BMI **(kg/m^2^)	0.313	>0.05	0.543	<0.01

**Hb **(g/dl)	0.498	<0.01	-0.408	<0.05

**Serum iron **(ug/dl)	0.478	<0.01	-0.375	<0.05

**TIBC **(mg/dl)	-0.411	<0.05	0.401	<0.05

**Transferrin **(umol/L)	-0.398	<0.05	0.398	<0.05

**Transferrin saturation **(%)	0.468	<0.01	-0.366	<0.05

**Ferritin **(ng/ml)	0.391	<0.05	-0.301	>0.05

P > 0.05 = non-significant, P < 0.05 = significant, P < 0.01 highly significant.(Pearson correlation analysis).

On the contrary, in obese children with IDA, serum hepcidin showed significant negative correlations with Hb, serum iron and transferrin saturation(P < 0.05) (Table 3). Otherwise it showed significant positive correlations with TIBC and transferrin (P < 0.05), (Table 3).

## Discussion

Recently, data from the American National Health and Nutrition Examination Survey III as well as data obtained in children from transition countries (Morocco and India) have suggested that among children, the prevalence of iron deficiency increases as BMI increases from normal weight to at risk for obesity to obesity [20-23]. It remains unclear, however, if the lower serum iron and elevated ferritin seen in obesity are most reflective of a functional iron deficiency related to an inflammatory state, or if obesity is also a risk factor for true iron deficiency [24].

At the beginning of our study, obese children with IDA had significantly higher serum ferritin levels than non-obese children with IDA (p < 0.01), and were similar to ferritin levels in healthy children (P > 0.05).

Obesity is considered a chronic inflammatory state [25]. Serum ferritin concentrations, which are usually suppressed when body iron stores are low [26], tend to be high and inversely related to transferrin saturation in those with excessive adiposity. Ferritin is considered an acute-phase reactant [24], hence, it may be elevated in inflammatory conditions even in the presence of true iron deficiency [26]. cytokines such as interleukin-1β and tumor necrosis factor-α (TNF-α) induce ferritin production within macrophages, hepatocytes and adipocytes [27].

In our study, obese children with IDA had significantly higher serum hepcidin levels (p < 0.01), in comparison to non-obese children with IDA and healthy control group (P < 0.01), in contrast, non-obese children with IDA had significantly lower serum hepcidin levels, compared to obese children with IDA and healthy control children (P < 0.01).

Hepcidin is a small peptide hormone secreted by the liver and by adipocytes [9]. Hepcidin is suppressed in iron deficiency, allowing increased absorption of dietary iron and replenishment of iron stores [28]. The feedback loop between iron and hepcidin ensures stability of plasma iron concentrations[29]. Hepcidin is an acute-phase reactant [10,24], and its expression is increased in chronic inflammatory states[30] including obesity [9]. Hepcidin can inhibit enterocyte iron absorption [31] and has further been shown to inhibit the release of non-heme iron from macrophages [32]. Because each of these actions diminishes the amount of bioavailable body iron, it has been suggested that when hepcidin is induced by inflammation, hepcidin is a key iron regulator that causes the hypoferremia and anemia of chronic disease [33].

In our study, CRP was significantly higher in obese children with IDA than in non-obese children with IDA and healthy control group (P < 0.05, respectively). Meanwhile, there was no significant difference between non-obese children with IDA and control group as regard CRP (p > 0.05). Yanoff et al [24] found that CRP concentrations were higher in obese subjects and were positively correlated with BMI, findings consistent with the observation that obesity is an inflammatory state that increases acute-phase reactants.

In our study, treatment of IDA by oral iron therapy for three months was associated with significant improvement of Hb, serum iron and TIBC in cases of IDA (p < 0.05), that was significantly more evident in non-obese children than obese children (P < 0.05).

Zimmermann et al [22] stated that adiposity in young women predicted not only lower iron absorption but also reduced response to iron supplementation, possibly due to increased hepcidin production.

In our study, serum ferritin increased significantly in non-obese children with IDA after 3 months of oral iron therapy (P < 0.01). On the other hand, obese children with IDA showed non-significant change in serum ferritin before and after oral iron therapy (P > 0.05).

In this study, serum hepcidin increased significantly in non-obese children with IDA after 3 months of oral iron therapy (P < 0.01) and reached to normal value in comparison to healthy control children (Figure 2). On the other hand, there was non-significant change in serum hepcidin after oral iron therapy in obese children (P > 0.01).

The regulation of hepcidin in adipose tissue remains unknown and may be similar to other adipokines in subcutaneous and epicardial adipose tissues. Inflammation-induced hepcidin stimulation is mediated through IL-6/STAT3 (signal transducer and activator of transcription-3) pathway [17]. On the other hand, mRNA for hemojuvelin; a surface molecule important for iron sensing and hepcidin production in the liver [34]; was not detected in adipose tissue. Hepcidin expression in adipose tissue is thus stimulated rather by inflammatory stimuli than by iron [35].

Bariatric surgery resulting in significant and long-lasting weight loss reduces inflammation and consequently, improves iron status in morbidly obese patients [36].

In the present study, serum hepcidin in non-obese children with IDA, showed significant positive correlation with Hb, serum iron and transferrin saturation (P < 0.01). In contrast, in obese children with IDA, serum hepcidin showed significant negative correlation with Hb, serum iron and transferrin saturation (P < 0.05).

Although liver hepcidin expression is *positively *associated with transferrin saturation, adipocyte hepcidin expression has a positive correlation with BMI, with a trend toward a *negative *association with transferrin saturation [9]. Therefore, lower bioavailability of iron among obese adults might be potentially related to the greater adipose hepcidin. Although hepcidin expression is more than 100-fold higher in hepatocytes than in adipocytes, secreted hepcidin from both tissues may have relevance for humans because in obesity, adipose tissue mass may be 20-fold greater than liver mass [24].

It is possible that the proinflammatory cytokines induced by the obese state increase hepcidin expression and upregulate ferritin synthesis in the reticuloendothelial cells [27] resulting in diminished absorption of iron in the setting of increased storage of iron, whether within the reticuloendothelial system or within adipocytes. Clinically, one would expect this to result in a combination of nutritional iron deficiency and functional iron deficiency [24]. The limitations of our study included inability to follow our studied obese children for a longer time to detect the possibility of delayed response to iron therapy and whether the reduction of weight will modify our results but that was due to lack of cooperation from the patients and their parents.

Finally, we can conclude that obesity increased hepcidin levels and was associated with diminished response to oral iron therapy in childhood iron deficiency anemia. Further studies in larger groups will be required to verify these findings and to assess the value of weight reduction in refractory IDA of obese children.

## Competing interests

The authors declare that they have no competing interests.

## Authors' contributions

MS participated in the design, collected samples and also participated in the analysis of data and discussion. MO, participated in the design and reviewed the results and discussion, AG conceived of the study and coordinated the sample collection. All authors read and approved all the manuscript.
